# Chronic Abdominal Pain: A Case of Giant Fecalith in the Distal Jejunum

**DOI:** 10.7759/cureus.7468

**Published:** 2020-03-30

**Authors:** Estrella Gutierrez, Diego Montelongo, Elizabeth Gamboa, Joseph Varon, Salim Surani

**Affiliations:** 1 Medicine, Dorrington Medical Associates, Houston, USA; 2 Medicine, Universidad Popular Autónoma del Estado de Puebla, Puebla, MEX; 3 Medicine, Dorrington Medical Associates, Texas, USA; 4 Medicine, Universidad Xochicalco, Ensenada, MEX; 5 Critical Care, United General Hospital, Houston, USA; 6 Critical Care, University of Texas Health Science Center, Houston, USA; 7 Internal Medicine, Texas A&M Health Science Center, Bryan, USA; 8 Internal Medicine, Corpus Christi Medical Center, Corpus Christi, USA; 9 Internal Medicine, University of North Texas, Dallas, USA

**Keywords:** fecalith, bowel obstruction, small intestine, jejunal fecalith

## Abstract

A fecalith is a mass of an accumulation of hardened fecal matter that is seen in patients with Chagas disease, Hirschsprung’s disease, and inflammatory bowel disease. In this article, we report a case of a 53-year-old female with chronic abdominal pain who was admitted with progressive weight loss, near syncope episode, and serum potassium of 2.6 mg/dL. An abdominal computed tomography (CT) scan revealed a left lower quadrant complex mass measuring 10.3 cm, with asymmetrical wall thickening and inflammatory stranding, non-discarding the compromise of the small bowel and consequent mild small bowel distention. A fecalith of 10.3 x 10.9 x 8.7 cm was found during an exploratory laparotomy in the small intestine. We report this rare case of distal jejunum fecalith accompanied by chronic pain.

## Introduction

In its extreme form, fecal impaction can lead to the formation of fecalith due to the hardening of fecal material that forms a mass separate from other bowel contents [1]. Most often, a fecalith can arise in the colon (mostly sigmoid, due to its smaller diameter) or rectum, and very rarely in the small intestine. The first case of a fecalith was described in 1967, where it was reported as a mass of hardened feces [2]. Our case is unique in that it highlights the importance of early detection of patients who are at risk and discusses a rare form of a small intestine fecalith.

## Case presentation

A 53-year-old female with a history of Crohn's disease, hypertension, thromboses, and an episode of intestinal obstruction presented to the emergency department in January 2020 with complaints of generalized weakness, progressive weight loss, near syncope a day prior, and abdominal pain 10/10 localized in both left and right upper quadrant.

The patient had a history of recurrent constipation, fast satiety and abdominal pain after eating or drinking water. The patient stated that she had weight loss of over 30 pounds over the past month, with a severe loss of appetite for the past six days. Upon her admission, she stated that abdominal pain had been presenting on and off for eight years, but it had exacerbated in the last four months. The pain lacked radiation and was accompanied by nausea and diarrhea. She had the need to stop her routine activities due to a progressive weakness episode which almost lead to very brief syncope; this event was accompanied by paresthesia in bilateral lower extremities and both hands clamping into a claw. The syncope was felt to be vasovagal related.

On physical examination, the patient was afebrile (temperature was 36.4˚C), had a heart rate of 83 beats per minute, blood pressure of 139/81 mmHg, respiratory rate of 17 respirations per minute with a saturation of 97%. Abdominal examination revealed a distention with areas of diffuse tenderness over the whole abdomen and decreased bowel sounds with no organomegaly noted.

**Figure 1:**
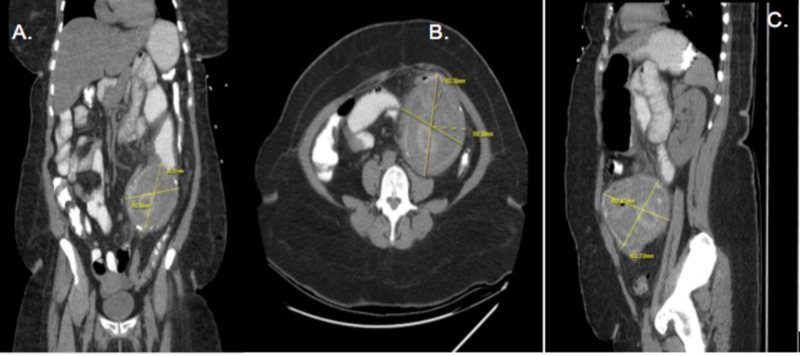
Computed tomography angiography (CTA) of the abdomen and pelvis with contrast showing complex mass with asymmetrical wall thickening, inflammatory stranding, and mild bowel distention (A) Axial view 10.33 cm x 10.03 cm, (B) Coronal view 10.3 cm x 9.32 cm and (C) Sagittal view 10.28 cm x 10.27 cm.

Laboratories showed hemoglobin of 11.0 g/dl, white blood cells 3.8x103/uL, and platelet count 354x103/uL. Among the renal function and serum electrolytes, the patient presented with severe hypokalemia with serum potassium of 2.6 mg/dL, creatinine of 0.7 mg/dL, sodium 133 mEq/L, magnesium 1.6 mg/dL, and phosphorous of 1.3mg/dL. Urinalysis revealed urinary tract infection and liver function tests were normal. Chest radiograph was unremarkable. The physician ordered a computed tomography (CT) scan of the abdomen which revealed a complex left lower quadrant mass measuring 10.3 cm with asymmetrical wall thickening, inflammatory stranding, probably involving the small bowel, and mild small bowel distention (Figure 1). The technical report included the following differential diagnoses: malignancy, abscess, and intussusception.

Colonoscopy resulted in no evidence of Crohn’s, it did however, show extensive colonic diverticulosis. Small bowel series ruled out inflammatory bowel disease or a ruptured diverticulum with inflammatory disease.

**Figure 2:**
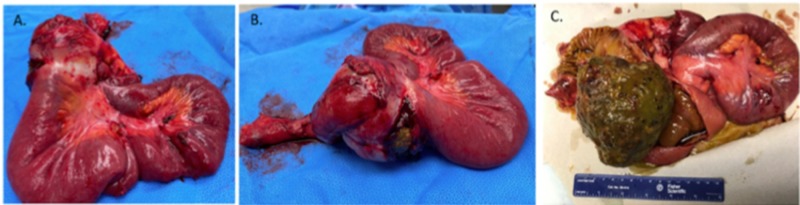
Resection of small bowel (A, B); fecalith (C)

**Figure 3:**
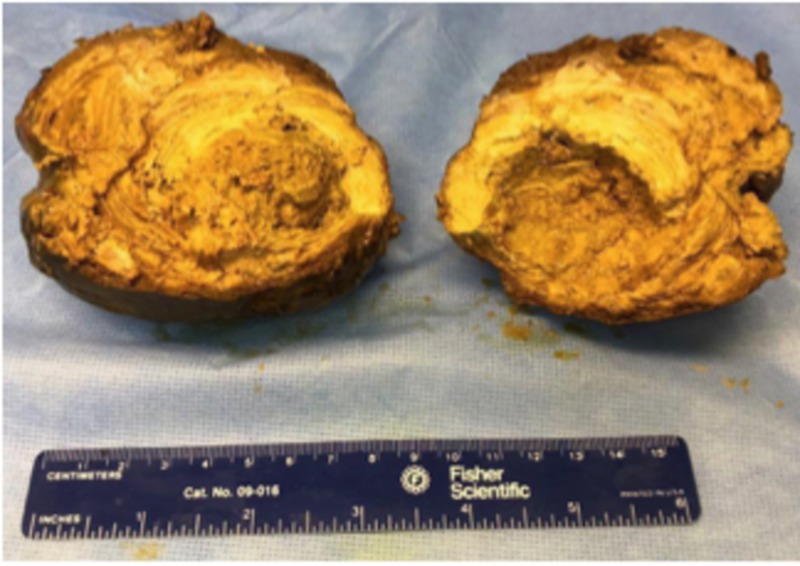
Resected fecalith measuring 10.3 x 10.9 x 8.7 cm, showing an inner hardened nucleus measuring 3.1 x 4 cm

The patient was scheduled for an exploratory laparotomy with excision for mass. Upon lysing the adhesions, the surgeon described a “large baseball sized mass” that was evident with the small bowel firmly attached to it. Mass was exposed and resected, which showed the fecalith (Figure 2). Our specimen was thoroughly examined post operation to confirm the absence of either upon dissection and examination, a multilayered 10.3 x 10.9 x 8.7 cm fecalith with no calcification and what appeared to be an inner hardened nucleus (Figure 3). 

The patient's post-operative course was unremarkable with good recovery. The patient was discharged home to be followed by their primary care physician as an outpatient. Per discussion with the primary care physician, the patient was doing well two weeks after discharge from the hospital.

## Discussion

Fecalith is the accumulation of hardened feces; it can form around a nidus, and even contain layers of calcifications [3]. Fecaliths, being rare per se, develop most commonly in the sigmoid colon and the descending colon; this is the second case report so far in the literature regarding a fecalith developing in the distal jejunum. When developing in the colon, the cause is a reduced diameter and harder stool consistency in the descending colon as compared to the ascending portion [3].

Interestingly, this patient had a fecalith in the small bowel, particularity the distal jejunum, which is an uncommon occurrence of an already rare phenomenon, with a single adult case previously reported in the English literature [3-4].

There is usually an associated disease, notably Hirschsprung, Chagas, inflammatory bowel disease, chronic constipation, or psychiatric diseases [5]. Nevertheless, the patient’s colonoscopy was negative for any Crohn’s manifestation, so this remains a question.

A fecalith acts like a giant fecal impaction and can cause obstructive, compressive, or luminal rupture [3]. Fecalith can give a broad spectrum of symptoms that present upon its location; patients with obstructive symptoms tend to have constipation, overflow diarrhea, and abdominal discomfort, while compressive symptoms can occur when the fecalith behaves like a tumor [3]. Additionally, this intraluminal mass can compress the ureters, causing hydronephrosis, and the bladder, causing urinary symptoms. Moreover, compression of the bladder can lead to rupture of the bladder wall, the uterus, the adjacent nerves, or nearby veins causing deep venous thrombosis. Without treatment, the fecaloma can rupture into the peritoneum or can cause the formation of an abscess.

There are cases of fecaliths that had an unfavorable outcome, leading to the death of the patient [6-7]. For this reason, we highly recommend practitioners to keep a high level of suspicion to avoid delay in diagnosis.

Conservative treatment may be attempted, but if it fails, the patient should undergo a surgical approach. Laparoscopies have been successfully attempted and if it fails, or if patient is not a suitable candidate for laparoscopy, then open laparotomy should be considered [8-9].

In regard to this case, having a fecalith in the small intestine eliminates any chance of a conservative approach being successful, thus, exploratory laparotomy followed by enterotomy was performed. Our case is rare due to the location of the fecalith, the clinical presentation and the evolution time of it.

## Conclusions

With nonspecific symptoms and a wide variety of differential diagnoses, a conservative approach to rule out the clinical suspicion of obstructive masses may be attempted. Surgeries such as laparotomy and laparoscopies provide a diagnostic and therapeutic option. In conclusion, fecalith in the small intestine is extremely rare, yet, it should be considered in the differential diagnosis when no proven intraluminal cause of chronic mechanical obstruction is encountered.
